# An Unexpected Diagnosis of Second Primary Malignancy in a Breast Cancer Survivor: A Case Report

**DOI:** 10.7759/cureus.42819

**Published:** 2023-08-01

**Authors:** Emaan Haque, Ali H Mushtaq, Radwan Alkhatib, Hamed Alhusaini, Kausar Suleman

**Affiliations:** 1 Department of Medicine, Alfaisal University, Riyadh, SAU; 2 Department of Medicine, Cleveland Clinic, Cleveland, USA; 3 Department of Oncology, King Faisal Specialist Hospital and Research Centre, Riyadh, SAU

**Keywords:** case report, second primary malignancy, neoplasms, lung adenocarcinoma, breast cancer

## Abstract

Background: Breast cancer survival rates are increasing more than ever with the development of better diagnostic and therapeutic techniques. Survivors of breast cancer have an increased risk of developing second primary malignancies, which may be mistaken for breast cancer recurrence and lead to delayed diagnosis and poor prognosis.

Case report: We report a case of a 62-year-old female who presented with shortness of breath and bone pain. She had a history of left triple-positive invasive ductal carcinoma (T1N0M0) treated with bilateral skin-sparing mastectomy, adjuvant Taxotere, and trastuzumab-based therapy and then continued on trastuzumab and letrozole. She underwent imaging to explore the source of her symptoms at which new pulmonary nodules were discovered. During workup, she was found to have elevated tumor markers. They were initially suspected to be breast cancer recurrence metastases based on elevated tumor markers; however, further investigations confirmed that the nodules were a second primary lung adenocarcinoma with a different molecular profile. The patient had disease progression despite chemotherapy and eventually succumbed to her disease.

Conclusion: This case highlights the importance of considering second primary malignancies in breast cancer survivors and utilizing advanced diagnostic modalities to efficiently diagnose such cases.

## Introduction

Breast cancer is the most common cancer in women in the US [1], with incidence rates highest in White women (133.7 per 100,000), followed closely by Black women (127.8 per 100,000) [2]. Fortunately, the survival rates for breast cancer have dramatically increased over the past several decades as a result of improvements in early detection and management, with the five-year survival rate improving from 75% for patients diagnosed in the mid-1970s to 90% for those diagnosed during 2011 through 2017 [3]. However, with increased survivorship, patients may also face a number of long-term sequelae such as being diagnosed with a second primary cancer. It can be difficult to diagnose these second primary malignancies in breast cancer survivors because they might be mistaken for the more frequent metastatic recurrence of the disease [4]. The prognosis and treatment choices for the two must be distinguished since they are distinct entities. However, due to their overlapping clinical characteristics and imaging results, this could prove to be challenging.

In patients with a history of breast cancer, pulmonary lesions are typically metastatic recurrences from the primary disease. However, albeit rare, these lesions can sometimes also be a distinct second primary lung cancer [5]. In this case report, we present a rare case of a breast cancer survivor who developed a second primary lung adenocarcinoma that was initially assumed to be a metastatic recurrence of breast cancer. We also describe the clinical course, diagnostic challenges, and management of this case and discuss the implications for clinical practice.

## Case presentation

**Figure 1:**
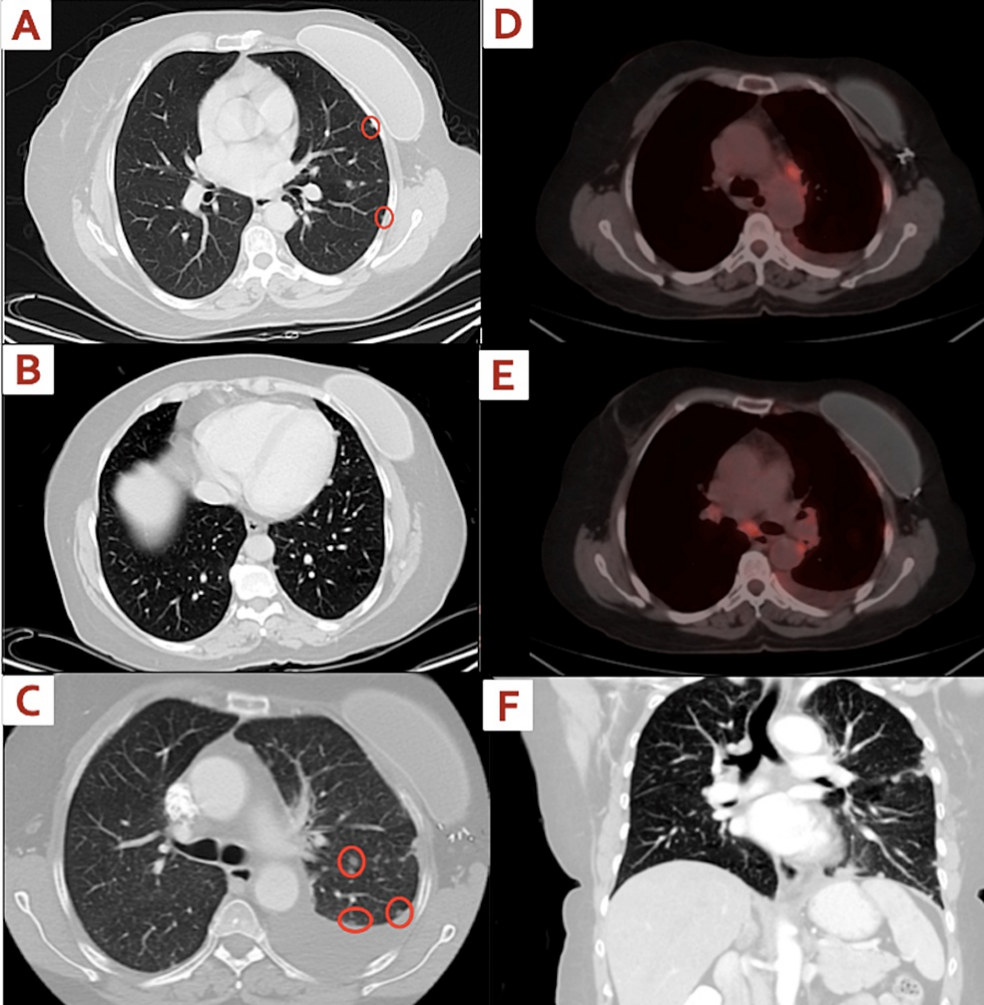
Imaging of nodules (A-C) Chest CT mediastinal window in the transverse plane from April 2016 showing subcentimeter left subpleural lung nodules. (D-E) Chest PET scan with FDG-avid left-sided pleural-based lung nodules. (F) Chest CT scan of the lung window in the coronal plane showing left upper lobe nodule involvement

We present the case of a 62-year-old non-alcoholic, non-smoker postmenopausal Arab woman who was diagnosed with a second primary lung adenocarcinoma four years after being initially diagnosed with stage I (T1N0M0) triple-positive (ER, PR, and HER-2/neu) invasive ductal carcinoma of the left breast. She had a strong family history of breast cancer with her sister and three of her first cousins also affected by the disease. At the time of her initial diagnosis of breast cancer, she underwent bilateral skin-sparing mastectomy, sentinel lymph node biopsy, and then adjuvant chemotherapy consisting of Taxotere and trastuzumab as well as hormonal therapy with letrozole for about four years. The patient maintained regular follow-up at our clinic and received routine blood work, which also included CA 15-3 measurements. Four years after her initial breast cancer diagnosis, the patient was found to have incidentally increased values of CA 15-3, although clinically asymptomatic. These rising values triggered a CT scan (Figure 1) to be performed which revealed multiple sub-centimeter lung nodules that were suspicious of malignancy. Additionally, an FDG-PET scan (Figure 1) showed FDG-avid left-sided lung nodules and left-sided pleural-based nodules with SUV measurements ranging from 6.7 to 4.2. Due to the small size of the lung nodules and the patient’s lack of symptoms, a decision was made to closely monitor the nodules and forego a bronchoscopic biopsy due to the potential risk of pneumothorax.

The patient decided to get a second opinion and, within a few weeks, was seen at a tertiary care cancer center in the US. However, by the time she reached there, her disease had progressed, and she had new liver and bone lesions along with worsening lung lesions. A biopsy of the liver lesions was performed; however, the procedure did not yield a substantial amount of sample tissue. Nevertheless, the sample was sent for next-generation sequencing analysis, while pathological analysis of the tissue revealed malignant cells suggestive of primary breast cancer origin that were ER and PR positive but HER-2 negative. It was speculated that the patient’s prior treatment with trastuzumab during her initial breast cancer diagnosis four years ago may have led to the loss of HER-2 positivity in the liver tissue biopsy sample. Hence, the patient was diagnosed with a metastatic recurrence of breast cancer to the lung, bone, and liver, and consequently, she was brought back to our institution in Saudi Arabia and switched from adjuvant letrozole to fulvestrant and palbociclib for treatment.

**Figure 2:**
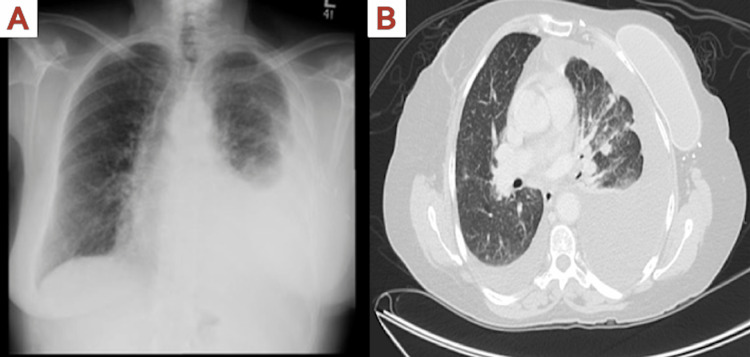
Imaging of pleural effusion (A) Chest X-ray showing pleural effusion. (B) CT in transverse plane showing pleural effusion

**Figure 3:**
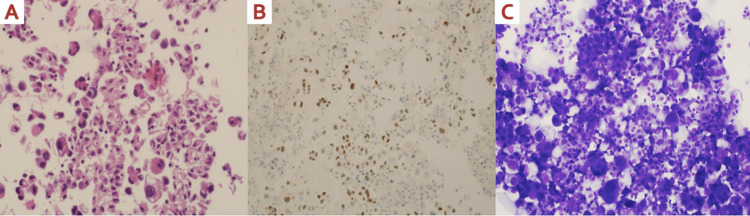
Pathology slides (A) Histopathology slide of the liver lesions taken from our patient showing abundant cytoplasm, prominent and pleomorphic nuclei with coarse chromatin pattern, and glandular or squamous architecture. (B) Immunohistochemical analysis of the liver biopsy shows TTF-1 and Napsin positive lesions confirming metastatic adenocarcinoma. (C) Pleural fluid cytology from thoracentesis shows malignant tumor cells that are also TTF-1 positive

After two months of treatment, the patient expressed concerns about worsening shortness of breath, and chest imaging revealed pleural effusion (Figure 2) for which she underwent diagnostic and therapeutic thoracocentesis along with biopsy of the lung nodules. Unexpectedly, the cytology analysis from the thoracocentesis revealed TTF-1 positive malignant cells consistent with lung adenocarcinoma (Figure 3). A re-evaluation of the prior liver pathology also found the sample to be positive for TTF-1 and Napsin (Figure 3). Moreover, the results of the next-generation sequencing analysis of the tissue sample sent earlier from the tertiary care cancer center in the US came back and revealed an epidermal growth factor receptor (EGFR) mutation with exon 19 deletion. These findings provided conclusive evidence that the patient had a second primary malignancy of stage IV lung adenocarcinoma with malignant pleural effusion as well as liver and bone metastasis. Additionally, these findings explained the reason behind the loss of HER-2 positivity observed in the initial liver biopsy.

Consequently, the treatment plan was modified to target EGFR-mutated non-small cell lung cancer. She was then stopped on the fulvestrant and palbociclib and then started on erlotinib, a tyrosine kinase inhibitor (TKI), along with denosumab for the bone metastasis, and palliative radiation to the spine from T11-L1. On follow-up, the patient had radiologic improvement in the metastatic pulmonary nodules and bone metastasis as well as clinical improvement of symptoms; however, her CA 15-3 levels continued to rise, reaching up to 144 U/mL. Interestingly, when the patient requested to continue treatment for her past breast cancer, in conjunction with treatment for her current lung cancer, and was started on the adjuvant aromatase inhibitor Exemestane, her CA 15-3 significantly dropped to 29.5, although this is primarily a treatment for breast cancer and not lung cancer. However, exemestane was later stopped due to intolerable side effects of dizziness and fatigue.

Despite the patient initially having radiological improvement in disease while on erlotinib, a CT scan conducted eight months after initiating treatment revealed disease progression in the lung, liver, and bone. Hence, a plasma EGFR test was performed and revealed a new T790M mutation of EGFR, conferring resistance to first-generation TKIs. The patient was then switched from erlotinib to the third-generation TKI osimertinib to treat the T790M-mutated EGFR tumor. Additionally, denosumab was added to treat bone metastasis.

The patient remained clinically and radiologically stable on this treatment regimen for about 14 months until she started experiencing significant deterioration in her mental status. Intracranial imaging revealed leptomeningeal thickening, consistent with brain metastasis; however, a subsequent lumbar puncture did not show any evidence of metastatic disease. Further neurologic workup for paraneoplastic syndromes yielded negative results as well. Given the patient’s poor performance status, a discussion was held with the patient’s family to assign a "do not attempt resuscitation" order and admit her to palliative care. Unfortunately, the patient succumbed to her disease while receiving inpatient palliative care approximately one year later, around three years after the initial diagnosis of lung cancer.

## Discussion

This case report presents a peculiar and challenging situation in which a breast cancer survivor developed a second primary lung adenocarcinoma with distinct molecular features and therapeutic responses. Differentiating between metastatic breast cancer and second primary lung cancer can pose a challenge, especially when the lung lesions are small and difficult to biopsy. Additionally, the presence of tumor marker CA 15-3, which can be elevated in both lung and breast cancer [6,7], further complicates the diagnostic process. Therefore, if feasible, it is crucial to obtain histological and molecular confirmation of the lung lesions since it may have a significant impact on the patient's prognosis and course of therapy.

Breast cancer patients who have surpassed 10 years of survival have about a 10% risk of developing a second primary malignancy, with lung cancer comprising approximately 5% of those cases [8]. Several variables, including age at diagnosis, time since diagnosis, family history of breast cancer, smoking, hormone receptor status, and treatment choices could influence the likelihood of developing a second primary lung cancer following breast cancer [9].

In this case, the patient had a number of risk factors for developing second primary lung cancer, including older age at breast cancer diagnosis (>50 years old) and a strong family history of malignancy [10]. Although previous studies have suggested a higher incidence rate of second primary lung cancers among patients with ER, PR, and HER-2 negativity [10-12], our patient had triple positive breast cancer, suggesting a potential alternate pathway to the development of her lung cancer.

Due to the unconventional clinical presentation and diagnostic findings observed in our patient, it was difficult to make an accurate diagnosis early in the disease course with the only significant finding being her rising CA 15-3. While current guidelines do not endorse the use of CA 15-3 for monitoring breast cancer recurrence, our patient specifically requested it to be followed, and her unique situation lends credence to its utility in guiding treatment decisions.

An interesting observation in this case is the significant reduction in our patient’s CA 15-3 levels upon initiation of exemestane, an aromatase inhibitor used to treat ER-positive breast cancer. The interplay between the ER signaling pathway and lung cancer is an area of active research, and estrogen’s role in lung cancer carcinogenesis through EGFR activation has been established [13]. Studies have shown that anti-estrogen treatment in ER-positive breast cancer patients can lower lung cancer incidence and improve long-term survival in patients with lung cancer after breast cancer [14]. For instance, a Swedish study randomized 4128 postmenopausal breast cancer patients to either a two-year or five-year tamoxifen treatment regimen and found that the five-year tamoxifen treatment group had a reduced incidence of squamous cell and small cell lung cancer [15]. Further research is warranted to explore this relationship between anti-estrogen therapies and lung cancer after breast cancer.

Typically, breast cancer survivors with incidental discovery of multiple sub-centimeter pulmonary nodules on CT scans are presumed to have metastatic disease, even though these lung nodules are often benign in the majority of cases [16,17]. Surprisingly in our patient’s case, neither breast cancer metastatic recurrence nor benign disease was found, but in fact a second primary lung cancer. Additionally, due to the sub-centimeter size of the initially biopsied liver lesions, a sufficient tissue sample could not be obtained for adequate immunohistochemical staining. This, combined with the background assumption of the pulmonary lesions favoring metastasis rather than a second primary malignancy, led to the initial misdiagnosis of metastatic breast cancer recurrence. This highlights the importance of revising unclear findings to minimize the risk of misdiagnosis as well as the importance of implementing next-generation sequencing technologies to further aid in making an accurate diagnosis.

EGFR mutations are frequently observed in non-small cell lung cancer, and their prevalence varies depending on the geographic region. Patients in Asia tend to exhibit a prevalence of about 49%, while patients in Europe have a prevalence of about 13% [18]. As seen in our patient, the most common EGFR sub-mutations are exon 19 deletions, with a prevalence ranging from 40% to 67% [18]. This particular sub-mutation leads to hyper-activation of pro-survival and anti-apoptosis signaling pathways, thus conferring favorable outcomes in patients treated with EGFR-TKI drugs, like the first-generation TKI erlotinib [19]. Initially, our patient was initiated on erlotinib, but after eight months, resistance set in due to the acquisition of the T790M mutation in the EGFR gene, which occurs in approximately 50% of patients who become resistant to first- or second-generation EGFR TKIs [20]. Subsequently, the patient’s treatment was switched to osimertinib, a third-generation EGFR TKI that has demonstrated higher efficacy and safety compared to existing EGFR TKIs in advanced non-small cell lung cancer patients with T790M mutation [19].

## Conclusions

In summary, this case study illustrates a rare instance of a breast cancer survivor who developed a second primary lung adenocarcinoma, initially misinterpreted as a metastatic recurrence of breast cancer. The importance of considering second primary malignancies in breast cancer survivors is highlighted in this case. Moreover, it demonstrates the potential value of genetic analysis and targeted therapy for individuals with second primary lung cancer who have actionable mutations. By sharing this case, we aim to contribute to the knowledge base on survivorship care and encourage multidisciplinary collaboration to optimize patient outcomes.
